# Deglutition-Induced Atrial Tachycardia

**DOI:** 10.7759/cureus.18448

**Published:** 2021-10-02

**Authors:** Nesrine Farah, Catalina Trana

**Affiliations:** 1 Cardiology, Hôpital Riviera Chablais, Rennaz, CHE

**Keywords:** autonomic nervous system, palpitations, supraventricular tachycardia, atrial tachycardia, swallowing, deglutition

## Abstract

While bradyarrhythmia is the most common arrhythmia during deglutition, tachycardias are considered to be a very rare condition with approximately 50 cases documented worldwide. The subjects are usually men with no structural heart disease or gastrointestinal pathology, and symptoms may vary from palpitations to lightheadedness or syncope.

Management is based on adapting alimentary habits in combination with agents such as beta-blockers, calcium channel blockers, and class IA, IC, and III drugs. Radiofrequency catheter ablation offers a permanent cure in the majority of the reported cases.

We report the case of a 51-year-old male with swallowing-induced palpitations, corresponding to brief episodes of atrial tachycardia. Beta-blockers and calcium channel blockers were interrupted because of intolerance. Lifestyle measures with fractionated meals allowing small boluses significantly reduced symptoms. The patient was reticent to invasive measures.

## Introduction

Deglutition-induced atrial tachycardia (DIAT) was first described in 1926 by Sakai and Mori [1] and is considered a very uncommon condition. Its exact mechanism is unclear, and it has been supposed to be a multifactorial phenomenon [2]. Management may be challenging with a variable efficacy of the medical treatment. Radiofrequency catheter ablation can be a permanent solution but may present some technical limitations [3].

## Case presentation

We present the case of a 51-year-old male with no significant prior cardiac history evaluated for a five-month history of mild palpitations. Symptoms happen during deglutition of moderate to important quantities of liquid and solid foods. They last for about 15-20 seconds and never occur outside mealtimes. There was no chest pain, shortness of breath, dizziness, lightheadedness, or syncope. The patient denied any symptoms of heartburn, dysphagia, or odynophagia. He was a nonsmoker and did not consume alcohol or excess caffeinated beverages. As a relevant condition, we mention the history of cecal adenocarcinoma pT4b pN2a M0 treated with right hemicolectomy and adjuvant chemotherapy five years ago with complete remission since then. A complete oncologic and gastrointestinal workup revealed no signs of recurrence of the oncologic disease. Minor chronic gastritis and esophagitis are diagnosed on esophagogastroduodenoscopy, which are treated with pantoprazole 40 mg per day; however, there was no change in the patient’s symptoms.

On physical examination, his blood pressure was 139/88 mmHg, and his pulse was 60 beats/minute and regular. Cardiovascular examination revealed normal heart sounds without any rubs, murmurs, or gallops. The chest was clear on auscultation. Abdominal examination was unremarkable. There was no thyromegaly. The complete blood count results, thyroid function hormones, electrolytes, and kidney function were normal.

**Figure 1:**
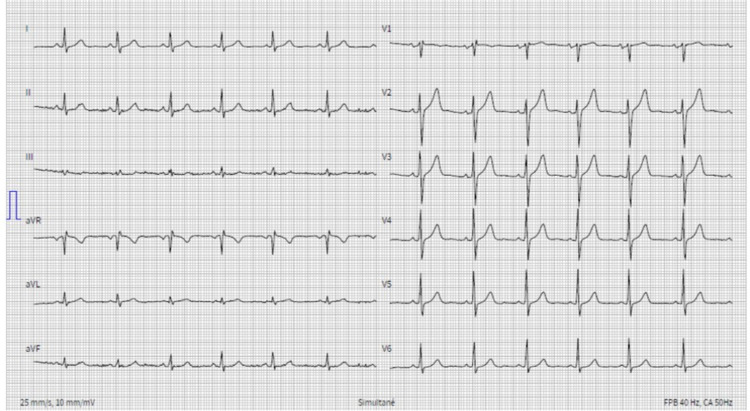
Resting 12-lead ECG showing sinus rhythm

Resting 12-lead ECG showed sinus rhythm and no abnormality (Figure 1).

**Figure 2:**
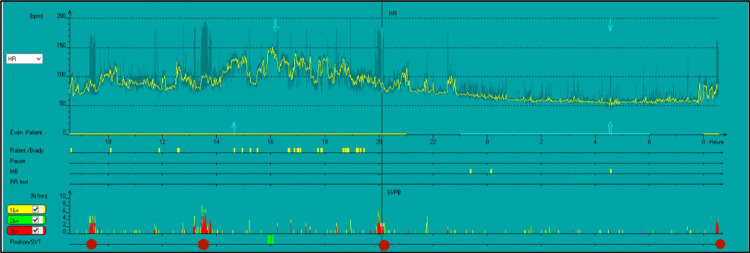
Twenty-four-hour Holter showing runs of DIAT during mealtimes (indicated with red dots)

**Figure 3:**
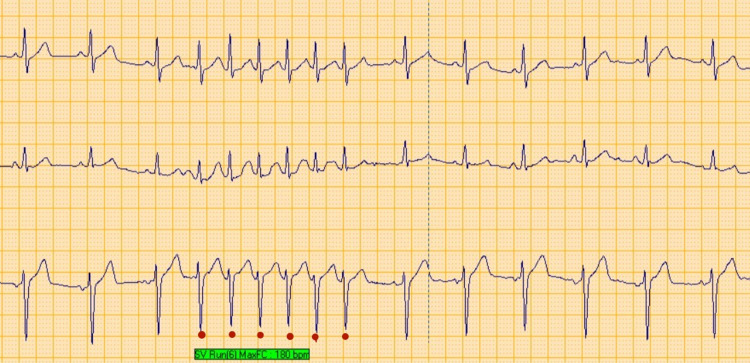
Twenty-four-hour Holter monitoring showing a nonsustained run of atrial tachycardia triggered by swallowing

Transthoracic echocardiography revealed normal left ventricular function, normal size of both left and right atrium, and no structural heart disease. A 24-hour Holter ECG monitor revealed brief runs of atrial tachycardia occurring at every episode of solid swallowing. Each episode lasts for 10-12 beats (Figures 2 and 3).

**Figure 4:**
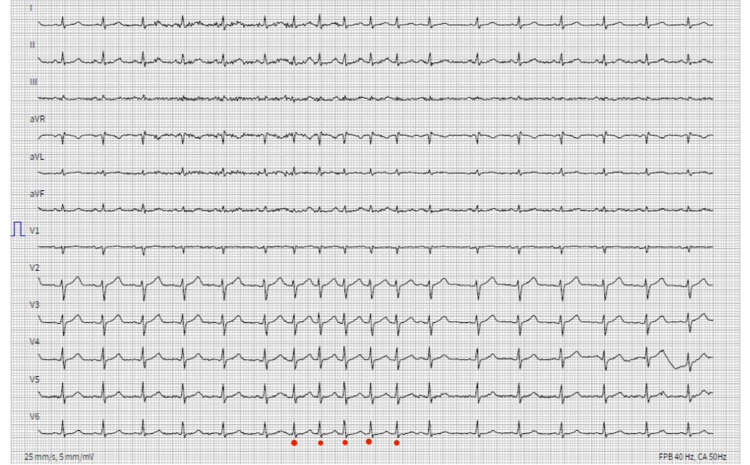
ECG monitoring during swallowing of chocolate

A continuous ECG monitoring in the office during swallowing of chocolate revealed also a nonsustained narrow complex tachycardia of five complexes (Figure 4).

The distinction of the p wave is critical during tachycardia, but it seems to occur before the QRS, i.e., RP > PR, denoting a long RP narrow QRS tachycardia. Possible diagnoses are atypical atrioventricular nodal reentrant tachycardia (AVNRT), permanent junctional reciprocating tachycardia (PJRT), atrial tachycardia, and sinus tachycardia. Atypical AVNRT and PJRT are excluded as the p wave is positive in inferior leads. The abrupt onset and termination of the episode exclude sinus tachycardia. Atrial tachycardia, more precisely sinoatrial nodal reentrant tachycardia (SANRT), a focal atrial tachycardia due to micro-reentry circuits arising from the sinus node, is the most likely diagnosis, owing to the p wave morphology.

A beta-blocker (metoprolol 25 mg and gradually 50 mg per day) was also started but subsequently stopped because of increased fatigue and depressive symptoms. The same issue was observed with calcium channel blockers (diltiazem R 90 mg twice per day). Both medications did not relieve symptoms, and the patient refused to undergo radiofrequency catheter ablation. Lifestyle measures by eating small food portions helped reduced the palpitations.

## Discussion

Dysrhythmias during food ingestion mostly refer to bradycardia with Mobitz type II atrioventricular block considered as the most frequent form [4]. They are thought to be a vagally induced phenomenon [5] and are associated with either an esophageal abnormality or a structural heart disease [6].

On the other hand, deglutition-induced tachycardia (DIT) is a very rare condition, generally observed in men without known heart or esophageal disease, and is mainly described as atrial tachycardia rather than true atrial fibrillation. Other possible DIT are atrial fibrillation, atrioventricular reentrant tachycardia, and atrioventricular nodal reentrant tachycardia. DIAT occurs only in 0.6% of patients who present with paroxysmal atrial arrhythmias [7].

The physiopathology of DIAT remains controversial, and many mechanisms were postulated [1,5,8]. The mechanical stimulation of the left atrium by a food bolus in the distended esophagus was supposed as a potential causative factor as Cohen et al. managed to reproduce atrial arrhythmias by inflating an intraluminal esophageal balloon at the subcarinal level [9]. However, as some DIAT were found to come from the right atrium in electrophysiologic studies, this mechanism is unlikely to explain all DIAT cases. Other potential mechanisms are the activation of the parasympathetic or the sympathetic autonomic system by swallowing. In fact, vagal stimulation known to cause bradycardia may also induce DIT by shortening the refractory period of atrial tissue in a disorganized way and inducing micro-reentrant circuits due to the dispersion of repolarization. This explains the effect of vagolytic agents such as atropine on some DIAT [2]. On the other hand, the stimulation of the cardiac sympathetic nervous system may also cause focal reentry circuits due to the alteration of the atrial depolarization and induce atrial arrhythmias [2,5,8]. This mechanism is supported by the efficacy of sympatholytic agents such as beta-blockers in stopping or reducing the duration of DIAT [2,8].

In our patient, the fact that the DIAT did not respond to the latter agents and get better with reducing food boluses may suggest a predominant mechanical phenomenon or vagal hyperstimulation as the most likely mechanisms. Further etiologic investigations were not performed upon the patient’s request.

Management of DIAT should be initially conservative with lifestyle changes consisting of fractionating food intake, opting for chopped foods, and minimizing caffeine, alcohol, ice, and cool liquid intake. The use of salbutamol, a beta 2 agonist, should be avoided [2]. The medications that are usually used are beta-blockers, calcium channel blockers, and class IA, IC, and III drugs [8]. Their efficacy is variable, depending on the predominant pathophysiological mechanism. Radiofrequency catheter ablation is considered to be the most effective solution for DIAT refractory to medical treatment. However, this procedure can be very challenging as the programmed electrical stimulation usually fails to induce the arrhythmia, and the mapping of the tachycardia during deglutition may cause aspiration on a patient under conscious sedation [8].

In our case report, no additional measures were attempted because of the mild nature of symptoms, already reduced with lifestyle change.

## Conclusions

DIAT is a rare condition resulting from various and sometimes interconnected mechanisms. The management may be laborious, but some cases present a good response to simple lifestyle measures.
